# Causal Relationship Between Electrocardiogram Parameters and Brugada Syndrome: A Bidirectional Mendelian Randomization Study

**DOI:** 10.1111/anec.70060

**Published:** 2025-03-12

**Authors:** Songcui Shen, Xiaolu Wang, Jingjuan Huang, Wenzhao Li

**Affiliations:** ^1^ Department of Cardiac Function Shanghai Chest Hospital, Shanghai Jiao Tong University School Of Medicine Shanghai China; ^2^ Department of Cardiology Shanghai Chest Hospital, Shanghai Jiao Tong University School Of Medicine Shanghai China

**Keywords:** bidirectional Mendelian randomization, Brugada syndrome, causal relationship, ECG parameters

## Abstract

**Background:**

Brugada syndrome (BrS) is associated with an electrocardiogram (ECG), but the causal relationship remains unclear. This study aimed to assess the bidirectional causal relationship between ECG parameters and BrS using Mendelian randomization (MR) analysis.

**Methods:**

A bidirectional MR analysis using data from the OpenGWAS database. Six ECG parameters, including PR interval, PP interval, ST duration, QRS duration, T wave duration, and QT interval, were included in the forward MR analysis with BrS as the outcome. In the reverse MR analysis, BrS was the exposure and the aforementioned ECG parameters were the outcomes. The inverse‐variance weighted (IVW) method was the primary analytical approach, complemented by four other methods to account for potential pleiotropy. Sensitivity analyses were performed using Cochran's Q test, MR‐Egger intercept, and leave‐one‐out analysis to evaluate heterogeneity and pleiotropy.

**Results:**

In the forward MR, genetically predicted ST duration (OR = 1.3478, 95% CI: 1.0611–1.7118, *p* = 0.014) and QRS duration (OR = 0.9582, 95% CI: 0.9208–0.9972, *p* = 0.036) showed significant associations with BrS. The reverse MR indicated that BrS was significantly associated with PR interval, QRS duration, P wave duration, and QT interval (all *p* < 0.05). Sensitivity analyses confirmed the robustness of the results in both forward and reverse MR analyses. However, there were significant horizontal pleiotropy and heterogeneity in reverse MR analysis.

**Conclusions:**

This MR study supported a causal effect of ECG parameters, including ST duration and QRS duration, on BrS development.

## Graphical abstract

This study uses Mendelian randomization to show that electrocardiogram parameters, including ST duration and QRS duration, have a causal effect on Brugada syndrome, offering new insights for understanding and managing the condition in clinical settings.

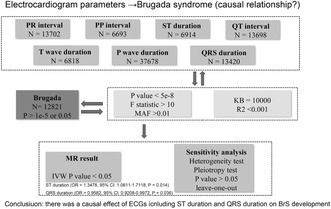

## 1 Introduction

Brugada syndrome (BrS) is an inherited cardiac disorder characterized by abnormal electrocardiogram (ECG) findings, which predispose individuals to arrhythmias and sudden cardiac death (Othieno et al. 2019). Although the exact pathophysiology of BrS has not been fully elucidated, studies have shown a strong association with mutations in sodium channel genes, particularly the SCN5A gene (Marsman et al. 2022). However, genetic predisposition alone is insufficient to fully explain the clinical manifestations of BrS. Other factors, such as ECG and structural cardiac changes, are also believed to play significant roles in the onset and progression of the disease (Brugada 2021; Isbister et al. 2023).

In studies of ECG parameters, patients with BrS typically present with characteristic ST‐segment elevation, increased J waves, and right bundle branch block (Krahn et al. 2022). These ECG parameters are not only used for the clinical diagnosis of BrS but also considered as potential disease markers (Vitali et al. 2021). However, the causal relationship between ECG markers and BrS has not been thoroughly investigated. Current studies are predominantly observational and are limited in their ability to fully exclude potential confounding factors, thereby constraining our understanding of the causal relationship between the two (Baena‐Palomino et al. 2024; Scrocco et al. 2024). Therefore, employing more rigorous methods to infer the causal relationship between ECG parameters and BrS remains a significant challenge in current research. Mendelian randomization (MR) is a causal inference method based on genetic variation that assesses the causal relationship between exposures and outcomes with reduced susceptibility to confounding factors and reverse causation (Huang et al. 2022). By using single‐nucleotide polymorphisms (SNPs) as instrument variables (IVs), MR provides a powerful tool for validating causal links between complex diseases and their potential risk factors. Existing studies have utilized MR to evaluate the causal relationships between ECG parameters and cardiovascular diseases. For instance, some MR studies suggest a potential causal relationship between ECG parameters and the occurrence of atrial fibrillation (Gajendragadkar et al. 2021). Other studies have indicated a close causal association between the PR interval and cardiac conduction system diseases (Hong et al. 2020). However, research on the causal relationship between ECG parameters and BrS remains relatively limited, particularly studies involving multiple ECG indices.

This study aimed to systematically evaluate the causal relationships between various ECG parameters and BrS using bidirectional MR methods, while also exploring the potential feedback effects of BrS on ECG parameters. The ECG parameters included in this study encompass various stages of cardiac electrophysiological activity, providing multidimensional data to support the investigation of the mechanisms underlying BrS. This research will not only enhance our understanding of the pathogenesis of BrS but also is expected to offer new theoretical insights into clinical screening, prevention, and treatment of BrS.

## 2 Methods

### 2.1 Data Sources

The data sources of ECG and BrS were taken from the OpenGWAS database (https://gwas.mrcieu.ac.uk/). The ECG data included PR interval, PP interval, ST duration, QRS duration, T wave duration, P wave duration, and QT interval. The detailed information for all enrolled data were listed in Table S1. In this study, forward MR was conducted with ECG parameters as the exposure and BrS as the outcome. Conversely, reverse MR was performed with BrS as the exposure and the various ECG parameters as the outcome. The flowchart was shown in Figure S1.

### 2.2 The Optimal IVs Selection

Appropriate IVs (SNPs) for ECG and BrS in current MR analyses were selected by using the TwoSampleMR package in R software (version: 4.3.2). Briefly, SNPs were selected at a threshold of genome‐wide significance (*p* < 5 × 10^−8^). Then, appropriate SNPs were kept based on linkage disequilibrium (LD) as measured by *r* ^2^ > 0.001 (clumping distance = 10,000 kb). When harmonizing exposure and outcome data, palindromic SNPs with intermediate allele frequencies were removed. Finally, F statistics were estimated to evaluate the instrument strength. In this study, the SNP with *F*‐statistic > 10 was considered a strong IV.

### 2.3 MR Analyses

The inverse‐variance weighted (IVW) method was primarily employed for fundamental causal estimates in current MR analyses, whereas the other four methods, including simple mode, weighted mode, weighted median, and MR‐Egger regression, were used to improve the IVW estimates as they could provide more robust estimates in a broader set of scenarios (Chen et al. 2022). If the *p* value of the IVW method was < 0.05, and the results of the other four methods were consistent in direction with the IVW result, the causal inference was considered statistically significant. The results were visualized using scatter plots and forest plots. Moreover, the MR‐Egger and IVW test were used to identify heterogeneity. The TwoSampleMR package in R was used for Cochran's *Q* analysis. For significant estimates, we further assessed horizontal pleiotropy using the MR‐Egger intercept test and MR‐PRESSO analyses. *p* < 0.05 was selected as the cutoff value for horizontal pleiotropy. Finally, the leave‐one‐out test was used for the sensitivity analysis.

## 3 Results

### 3.1 The Causal Effects of ECG on BrS

**FIGURE 1:**
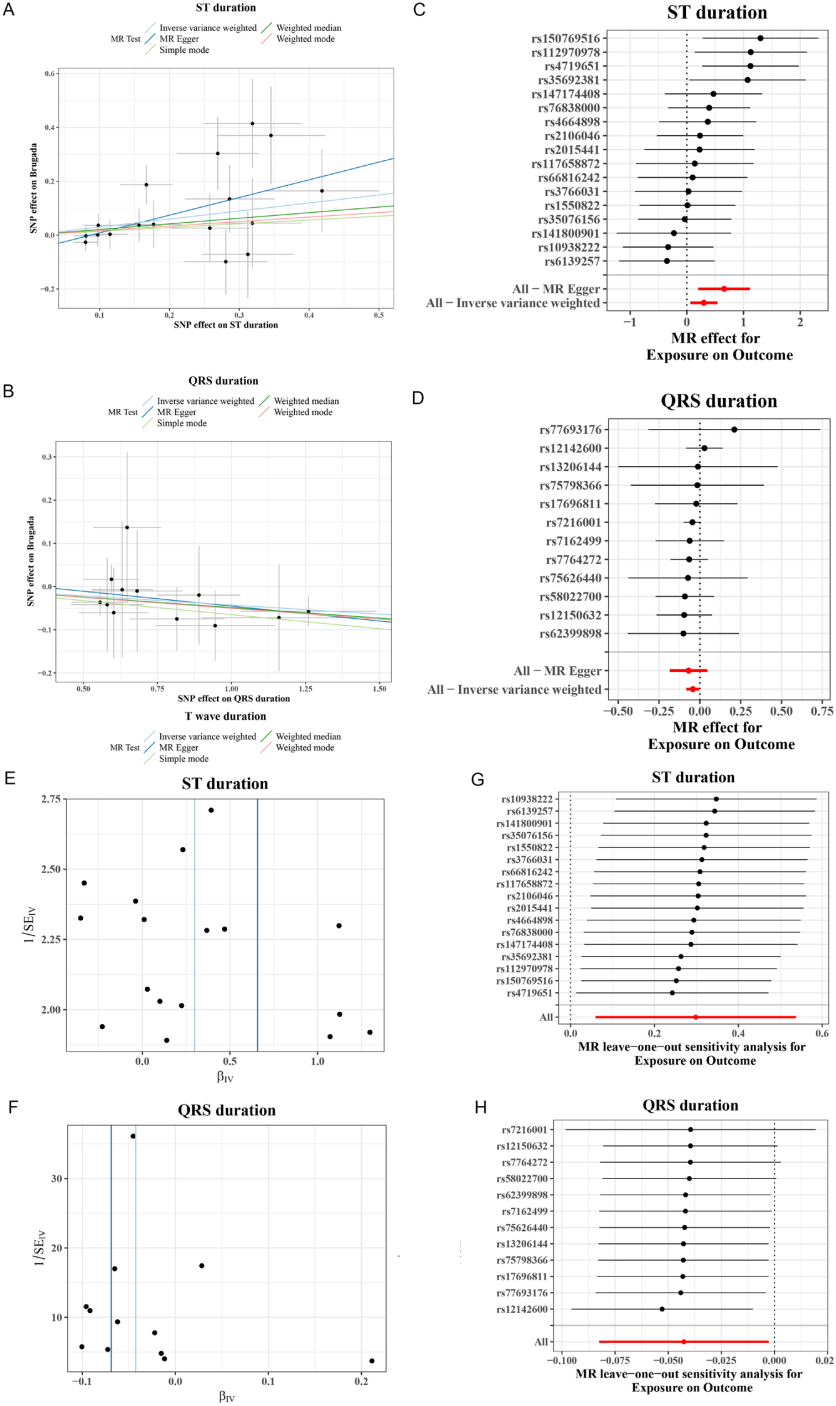
The forward Mendelian randomization (MR) analyses: Causal effect of Electrocardiogram (ECG) parameters on Brugada syndrome (BrS). (A, B) scatter plot of the causal relationships of ECG (ST duration and QRS duration) on BrS by using different MR methods: The slope of each line corresponds to the causal estimates for each method; individual SNP effect on the outcome (point and vertical line) against its effect on the exposure (point and horizontal line) was delineated in the background. (C, D) forest plot analysis for each SNP in ST duration and QRS duration: The inverse variance weighted (IVW) and MR‐Egger MR results are shown at the bottom. (E, F) funnel plot analyses to evaluate sensitivity for ST duration and QRS duration, respectively. (G, H) leave‐one‐out analyses to evaluate sensitivity: All SNP results are on the right side (ST duration) or left side (QRS duration) of 0, indicating that our analysis results are not sensitive.

There were no suitable SNPs of P wave duration enrolled for subsequent analysis after LD investigation; totally six ECG parameters, including PR interval (13 IVs), PP interval (10 IVs), ST duration (17 IVs), QRS duration (11 IVs), T wave duration (18 IVs), and QT interval (13 IVs), were used for forward MR analysis (Table 1). The IVW result suggested that genetically predicted ST duration (OR = 1.3478, 95% CI: 1.0611–1.7118, *p* = 1.44E‐2) and QRS duration (OR = 0.9582, 95% CI: 0.9208–0.9972, *p* = 3.62E‐2) were significantly associated with BrS (Table S2). The scatter plot analysis of different MR tests on ST duration and QRS duration was shown in Figure 1A,B, respectively. The detailed related information for all ECG parameters was shown in Figure S2. By combining the *p* value of Cochran's Q in MR‐Egger regression and IVW methods, it was suggested that the observed association was along without obvious heterogeneity (all *P* of Cochran's *Q* > 0.05) (Figure 1C,D). The detailed related information for all ECG parameters was shown in Figure S3. Meanwhile, there was no evidence of horizontal pleiotropy across SNPs in the causal estimates by MR‐Egger regression (Table 1). Subsequently, the funnel plot analyses and leave‐one‐out were used to conduct sensitivity on IVs. The funnel plot showed a relatively uniform dispersion of points, which further proved that our analysis results were not sensitive (Figure 1E,F). The detailed related information for all ECG parameters was shown in Figure S4. Even if removing any single SNP, the observed association did not significantly change in leave‐one‐out analyses (Figure 1G,H), suggesting the stability of the results. The detailed related information for all ECG parameters was shown in Figure S5.

**TABLE 1:** The results of sensitivity analysis in forward MR analysis.

Outcome	Exposure	Method	Heterogeneity test			Pleiotropy test		MR presso
			*Q*	*Q*_df	*Q*_*p*val	Egger intercept	*p*	*p*
Brugada	PR interval	MR Egger	13.6698	11	0.2518	−0.0352	0.3237	0.2290
Brugada	PR interval	Inverse variance weighted	14.9961	12	0.2416	NA	NA	
Brugada	PP interval	MR Egger	7.0511	8	0.5311	−0.0193	0.6914	0.6170
Brugada	PP interval	Inverse variance weighted	7.2206	9	0.6142	NA	NA	
Brugada	ST duration	MR Egger	16.4046	15	0.3557	−0.0575	0.0978	0.2430
Brugada	ST duration	Inverse variance weighted	19.8148	16	0.2287	NA	NA	
Brugada	QRS duration	MR Egger	3.2535	10	0.9748	0.0236	0.6460	0.9847
Brugada	QRS duration	Inverse variance weighted	3.4777	11	0.9828	NA	NA	
Brugada	T wave duration	MR Egger	19.3809	16	0.2494	0.0113	0.7422	0.2990
Brugada	T wave duration	Inverse variance weighted	19.5167	17	0.2997	NA	NA	
Brugada	QT interval	MR Egger	13.6698	11	0.2518	−0.0352	0.3237	0.2550
Brugada	QT interval	Inverse variance weighted	14.9961	12	0.2416	NA	NA	

Abbreviation: MR, Mendelian randomization.

### 3.2 The Causal Effects of BrS on ECG

**FIGURE 2:**
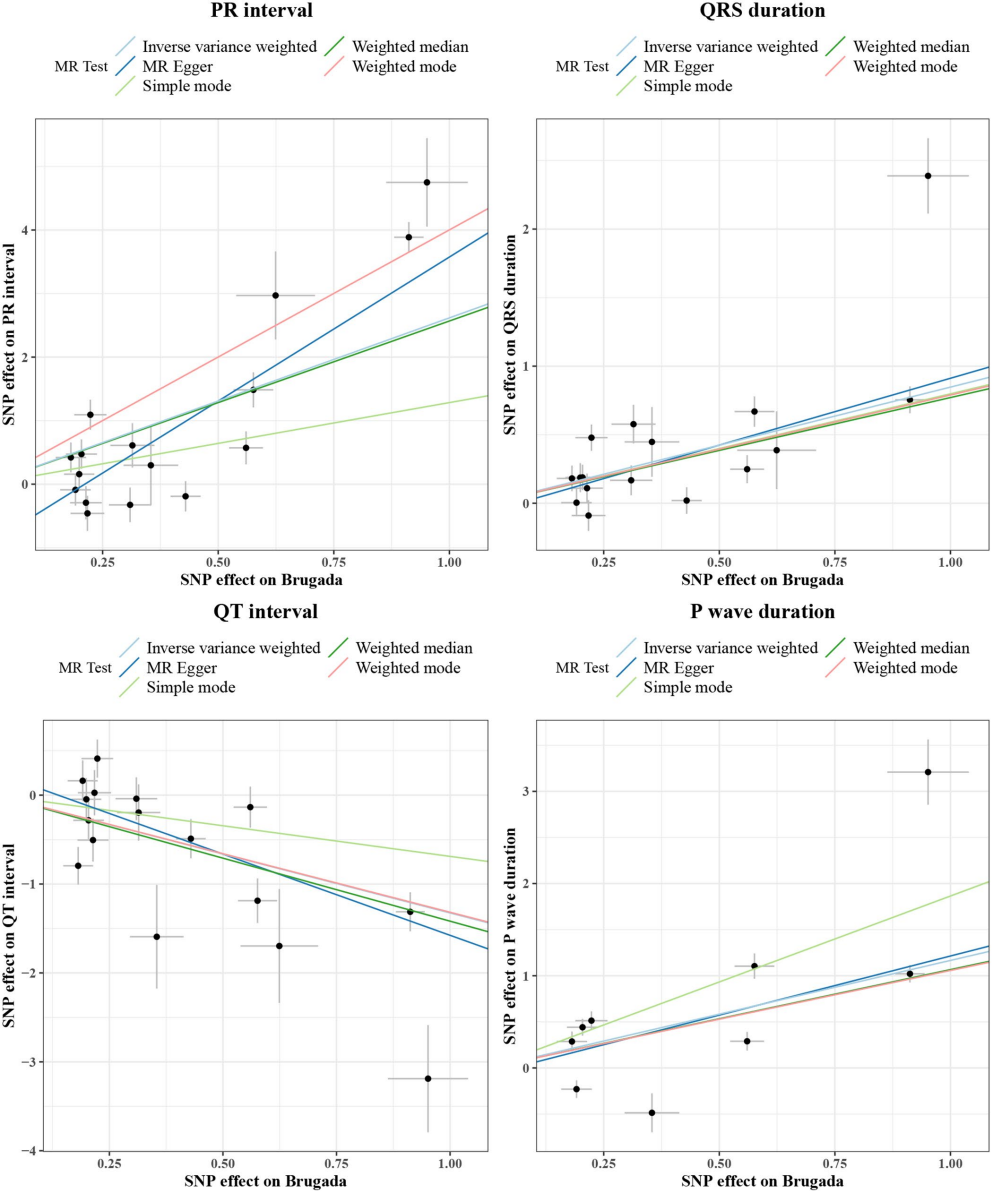
The scatter plot showed the causal relationships of BrS on ECG (PR interval, QRS duration, P wave duration and QT interval) using different MR methods. The slope of each line corresponds to the causal estimates for each method; the individual SNP effect on the outcome against its effect on the exposure was delineated in the background.

**FIGURE 3:**
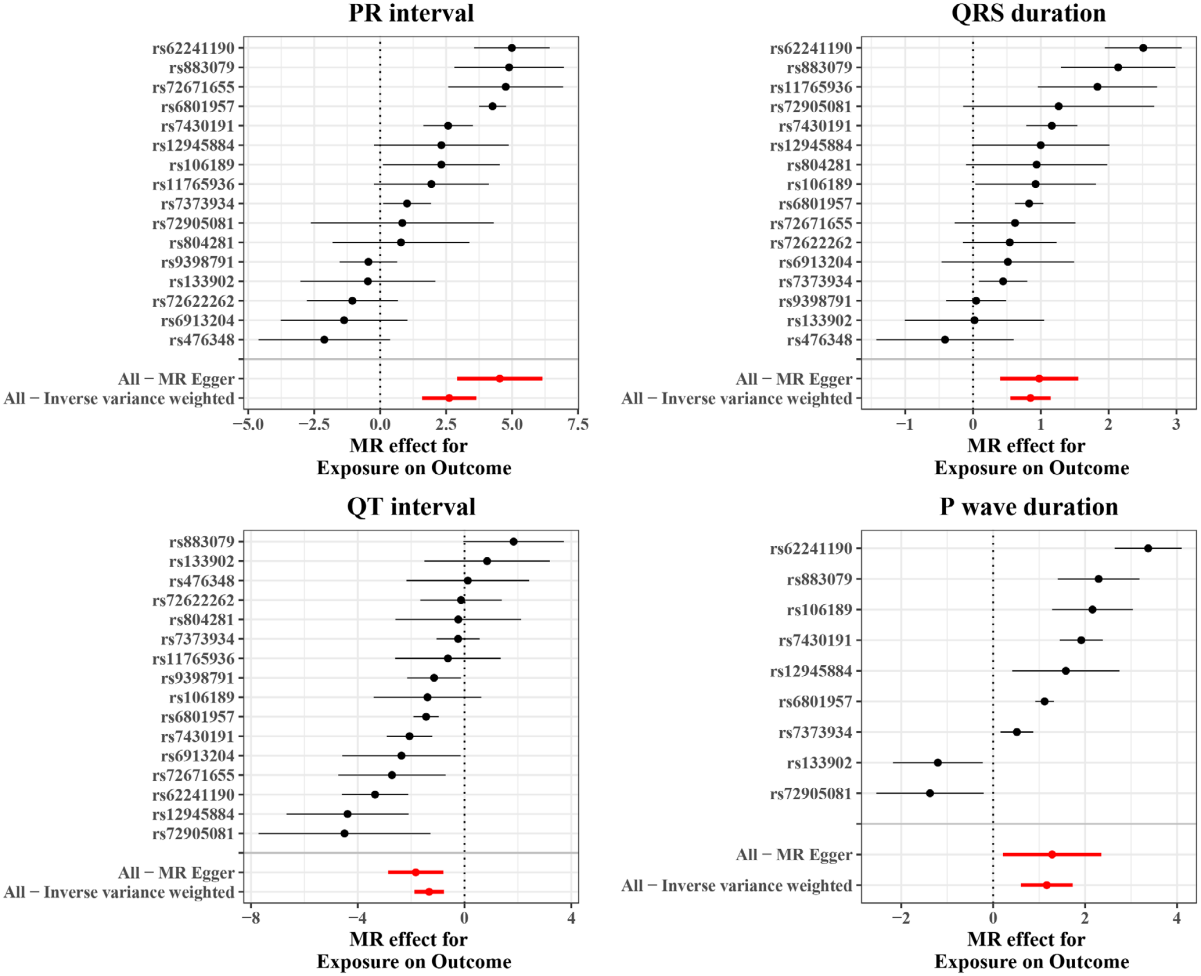
The forest plot analysis for each SNP in PR interval, QRS duration, P wave duration, and QT interval. The IVW and MR‐Egger MR results were shown at the bottom.

**FIGURE 4:**
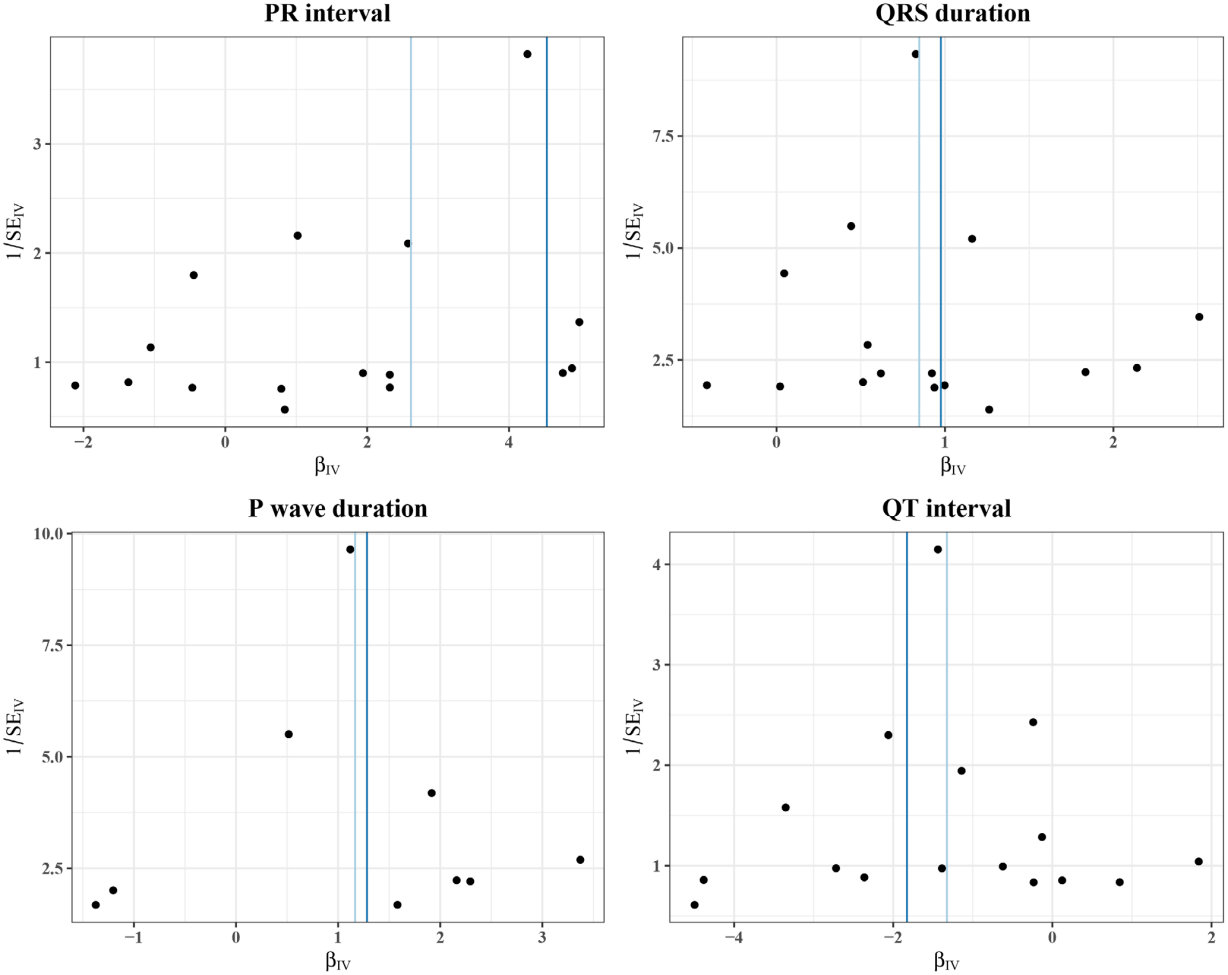
The funnel plot analysis to evaluate sensitivity for PR interval, QRS duration, P wave duration, and QT interval.

**FIGURE 5:**
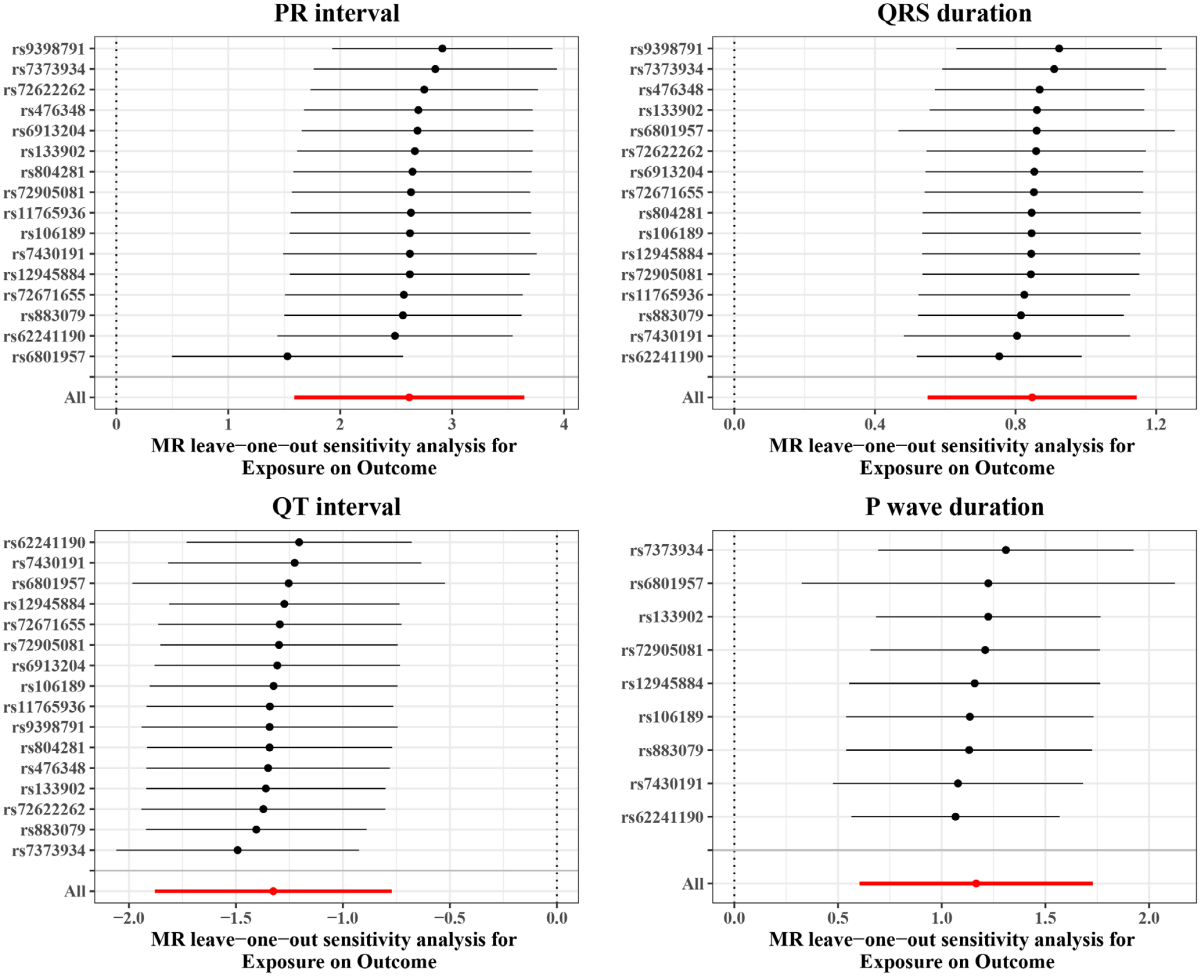
The leave‐one‐out analyses to evaluate the causal effect of BrS on ECG parameters including PR interval, QRS duration, P wave duration, and QT interval. Each point represented the effect estimate when one SNP was removed at a time, allowing assessment of the influence of each individual SNP on the overall causal estimate. All SNP results were on the right side (PR duration, QRS duration and P wave duration) or left side (QT duration) of 0, indicating that our analysis results were not sensitive.

Since no suitable SNPs were found for outcomes including P interval, ST duration, and T wave duration after LD investigation, in the reverse MR analysis, we incorporated 16 independent SNPs each for PR interval, QRS duration, and QT interval, and 9 independent SNPs for P wave duration as IVs. The IVW result suggested that genetically predicted BrS was significantly associated with PR interval (OR = 13.6996, 95% CI: 4.9019–38.2866, *p* = 5.9887E‐7), QRS duration (OR = 2.3331, 95% CI: 1.7329–3.1412, *p* = 2.3575E‐8), P wave duration (OR = 3.2097, 95% CI: 1.8285–5.6343, *p* = 4.8596E‐6), and QT interval (OR = 0.2657, 95% CI: 0.1527–0.4621, *p* = 2.6966E‐6) (Table S3). The scatter plot analysis of different MR tests on PR interval, QRS duration, QT interval, and P wave duration was shown in Figure 2. By combining the *p* value of Cochran's Q, it was suggested that the observed association was along with obvious heterogeneity (all *p* < 0.05) (Figure 3). Meanwhile, the horizontal pleiotropy was observed across SNPs in the causal estimates by MR‐PRESSO (all *p* < 0.01) (Table 2). Subsequently, the funnel plot analyses and leave‐one‐out were used to conduct sensitivity on IVs. The funnel plot showed a relatively uniform dispersion of points, which further proved that our analysis results were not sensitive (Figure 4). Even if removing any single SNP, the observed association did not significantly change in leave‐one‐out analyses (Figure 5), suggesting the stability of the results.

**TABLE 2:** The results of sensitivity analysis in reverse MR analysis.

Outcome	Exposure	Method	Heterogeneity test			Pleiotropy test		MR presso
			*Q*	*Q*_df	*Q*_*p*val	Egger_intercept	*p*	*p*
PR interval	Brugada	MR Egger	98.41997	14	9.52E‐15	−0.9585	0.015893	< 0.001
PR interval	Brugada	Inverse variance weighted	151.2746	15	1.35E‐24	NA	NA	
QRS duration	Brugada	MR Egger	76.1482	14	1.46E‐10	−0.06396	0.614424	< 0.001
QRS duration	Brugada	Inverse variance weighted	77.59215	15	1.92E‐10	NA	NA	
P wave duration	Brugada	MR Egger	109.449	7	1.19E‐20	−0.06848	0.80448	< 0.001
P wave duration	Brugada	Inverse variance weighted	110.4827	8	3.03E‐20	NA	NA	
QT interval	Brugada	MR Egger	48.60218	14	1.04E‐05	0.250791	0.281153	< 0.001
QT interval	Brugada	Inverse variance weighted	52.96463	15	3.91E‐06	NA	NA	

Abbreviation: MR, Mendelian randomization.

## 4 Discussion

Although BrS is an inherited cardiac disorder characterized by abnormal ECG findings (Bisignani et al. 2023), the causal relationship of ECG parameters on BrS is still unclear. In this bidirectional MR study, we found a causal effect of ECG parameters, including ST duration and QRS duration, on BrS, which further supported the association between ECG parameters and BrS.

One of the hallmark features of BrS is the characteristic ST‐segment elevation observed on ECGs, particularly in the right precordial leads (V1–V3) (Vinod and Patel 2024). This ST‐segment abnormality is closely associated with an increased risk of ventricular arrhythmias in BrS patients (Korlipara et al. 2021). Additionally, studies have shown that in some BrS patients, prolonged QRS duration may be linked to a higher risk of sudden cardiac death, further indicating that QRS prolongation could serve as a marker of abnormal myocardial electrical activity (Chakraborty et al. 2024). In our forward MR analysis, significant associations were found between genetically predicted ST duration and QRS duration with BrS. Specifically, the results suggested that increased ST duration was associated with a higher risk of BrS, while QRS duration was negatively associated with BrS. At the same time, heterogeneity and pleiotropy analyses were used in the analysis, and the results both showed that the forward MR analysis had no pleiotropic effect on the estimation of causality between the two and was not affected by a single SNP, which further enhanced the reliability of the results. These findings align with the well‐established role of abnormal ECG patterns in BrS, where prolonged ST duration is characteristic of the disease and linked to abnormal repolarization in the right ventricular outflow tract (Behr et al. 2021; Morita et al. 2008). The observed negative association between QRS duration and BrS may appear counterintuitive, given that prolonged QRS duration is often associated with various cardiac conduction disorders (Fabiszak et al. 2020). However, it may indicate a compensatory mechanism or reflect distinct electrophysiological characteristics specific to BrS. These findings corroborate with existing studies that emphasize the importance of ventricular depolarization and repolarization abnormalities in BrS pathogenesis (Pannone et al. 2022). Nonetheless, our results provided a novel causal insight by utilizing genetic instruments to validate the link between specific ECG parameters and BrS, which had previously been explored mainly through observational studies. These results could also suggest potential avenues for further investigation, such as exploring how modifying these ECG parameters might influence disease progression or prevention strategies for BrS.

In the reverse MR analysis, BrS was found to have significant causal effects on multiple ECG parameters, particularly PR interval, QRS duration, P wave duration, and QT interval. The most notable finding was the strong association between BrS and PR interval, which exhibited a marked increase in individuals genetically predisposed to BrS (Tadros et al. 2019). This suggests that the presence of BrS can causally lead to changes in atrioventricular conduction. These results echo findings from earlier studies, where PR interval prolongation was observed in BrS patients (Chung 2019; Martins de Carvalho et al. 2023), further supporting the idea that BrS not only affects ventricular depolarization but also disrupts atrioventricular node function. The association between BrS and prolonged QRS duration is consistent with its known impact on ventricular conduction, which has been previously highlighted in electrophysiological studies of BrS patients (Ohkubo et al. 2011). Interestingly, the negative causal effect of BrS on QT interval indicates a shortening of repolarization time, which may reflect the dynamic and complex interaction between different phases of the cardiac cycle in BrS patients. Thus, the reverse MR analysis indicated that BrS not only affected ECG parameters at the genetic level but also drove disease progression by altering these electrophysiological indicators. These findings provided new insights into the multifaceted pathophysiological mechanisms of BrS, particularly the central role of cardiac conduction and repolarization abnormalities. Future research should explore whether modulating these ECG parameters slows the progression of BrS or reduces the risk of sudden cardiac death in patients. However, there were still some limitations in the current study. First, despite efforts to control for horizontal pleiotropy, the complex genetic mechanisms underlying BrS may still pose challenges, and residual pleiotropy or other confounding factors cannot be entirely excluded. Additionally, certain ECG parameters, such as P wave duration, were not included in the forward MR analysis, potentially limiting our comprehensive understanding of the relationship between all electrophysiological traits and BrS. Moreover, although this study found a causal relationship between ST duration and QRS duration in the development of BrS, there is a lack of information on ST segment elevation in GWAS. ST‐segment elevation on ECG was previously reported to be a hallmark feature of BrS (Hartwig et al. 2024; McBenedict et al. 2024). This provides us with ideas for further analysis, and in the future, more data should be included to further verify that ST segment elevation is a hallmark of BrS and explore whether other electrocardiogram parameters that have a causal relationship with BrS can serve as a hallmark feature of BrS. Lastly, this study was based solely on European populations, and the findings may not be directly generalizable to other ethnic groups. Thus, a further study based on a larger, more diverse population is needed to validate these findings and improve the generalizability of the results.

In conclusion, this study provided novel insights into the bidirectional causal relationship between ECG parameters and BrS, highlighting the significant causal effect of ST duration and QRS duration on BrS development. These findings offer new perspectives for both understanding and potentially managing BrS in clinical settings.

## Author Contributions

All the authors have conceived and designed the article. Songcui Shen and Xiaolu Wang analyzed the data and wrote the article. Jingjuan Huang and Wenzhao Li collected the data and reviewed the articles.

## Conflicts of Interest

The authors declare no conflicts of interest.

## Supporting information


Appendix S1.

## Data Availability

The datasets generated during and analyzed during this study are available from the corresponding author upon reasonable request.
