# Supplementary material for: Causal Relationship Between Electrocardiogram Parameters and Brugada Syndrome: A Bidirectional Mendelian Randomization Study
Source: Ann Noninvasive Electrocardiol. 2025 Mar 12;30(2):e70060. doi: 10.1111/anec.70060 (PMC11897611; doi:10.1111/anec.70060)
Supplement: Supplementary file 1 — Appendix S1. [file ANEC-30-e70060-s001.docx]

**
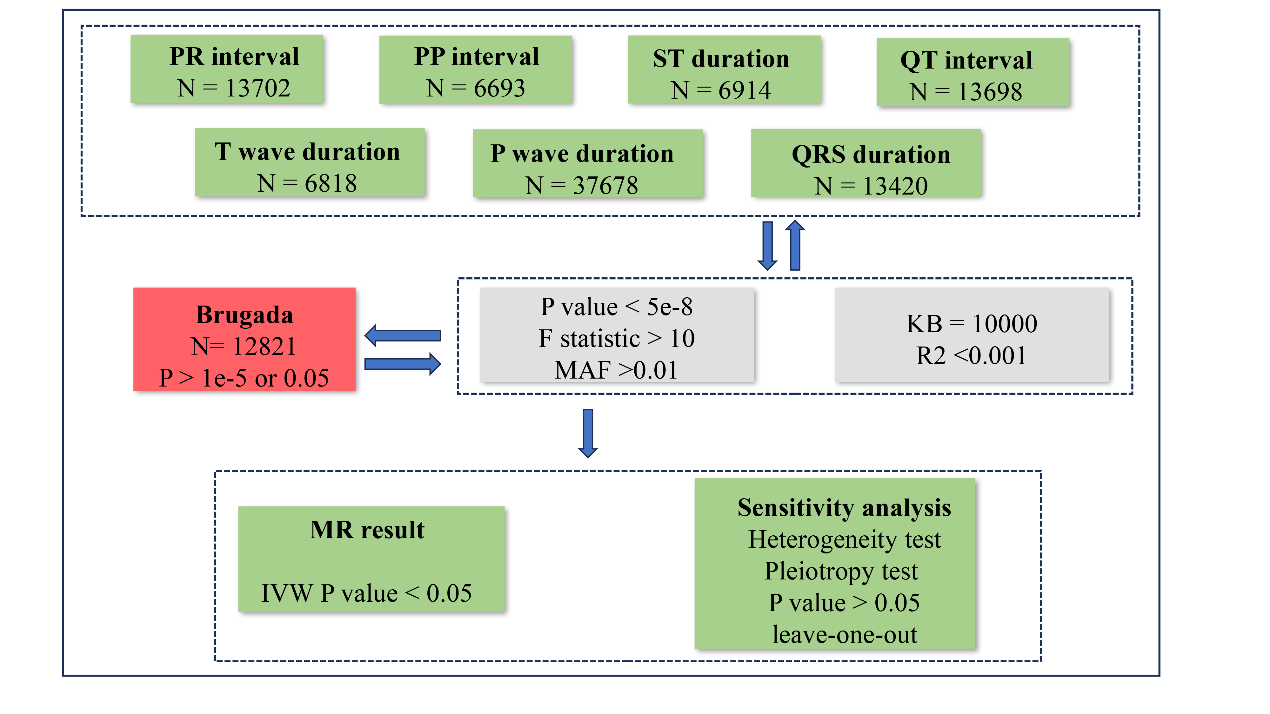
**

**Supplementary Figure 1 The flowchart for current MR analysis.**

**
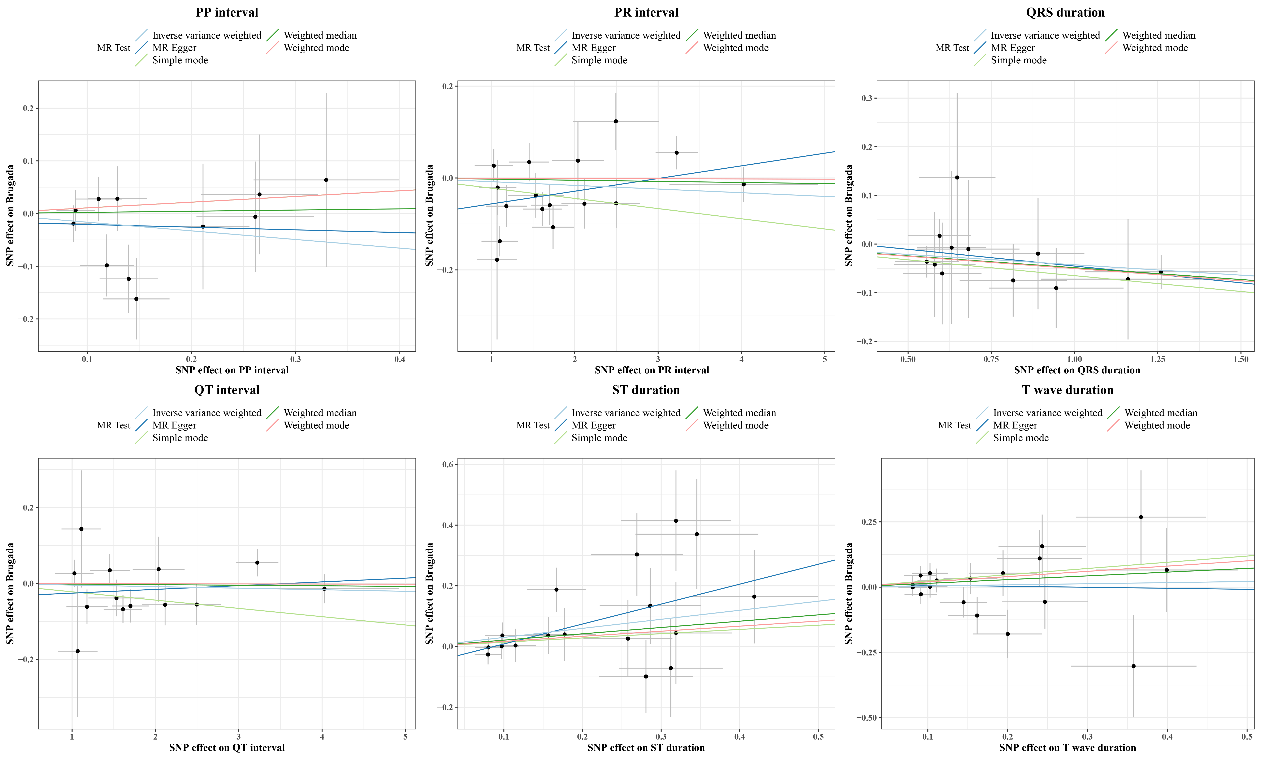
**

**Supplementary Figure 2 The causality scatter plot for forward MR analysis.**

**
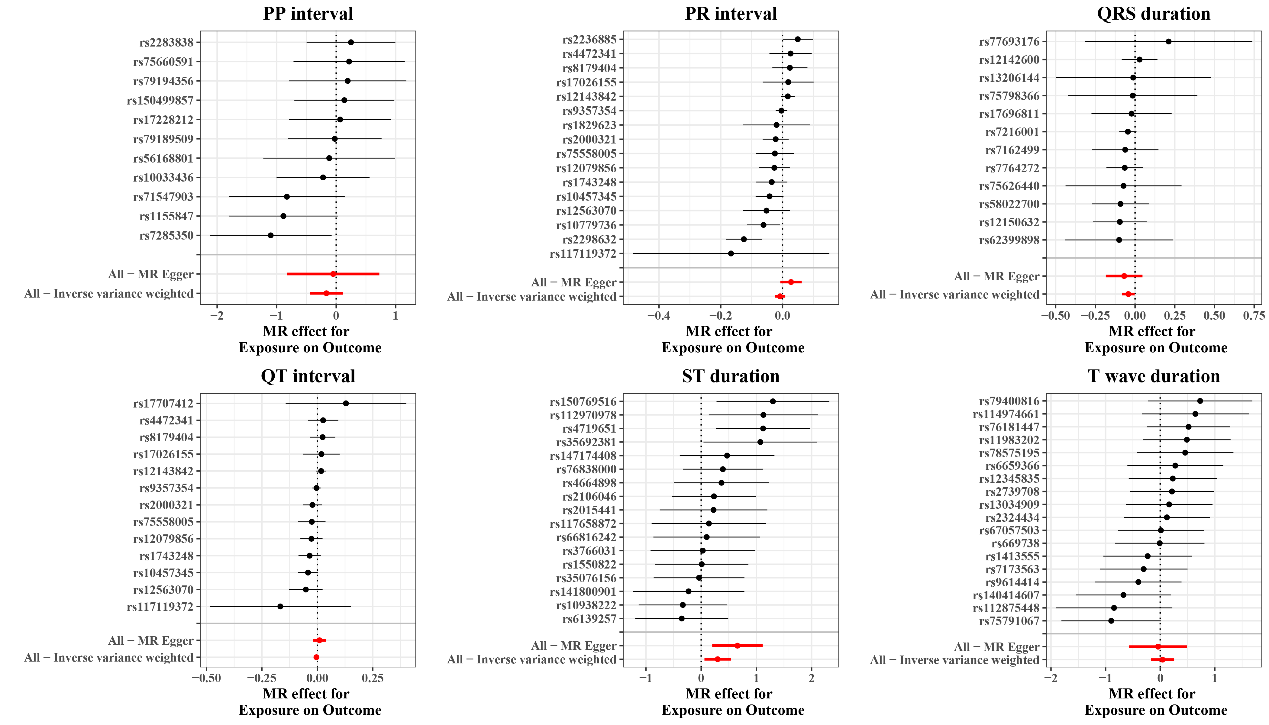
**

**Supplementary Figure 3 The forest plot for forward MR analysis.**

**
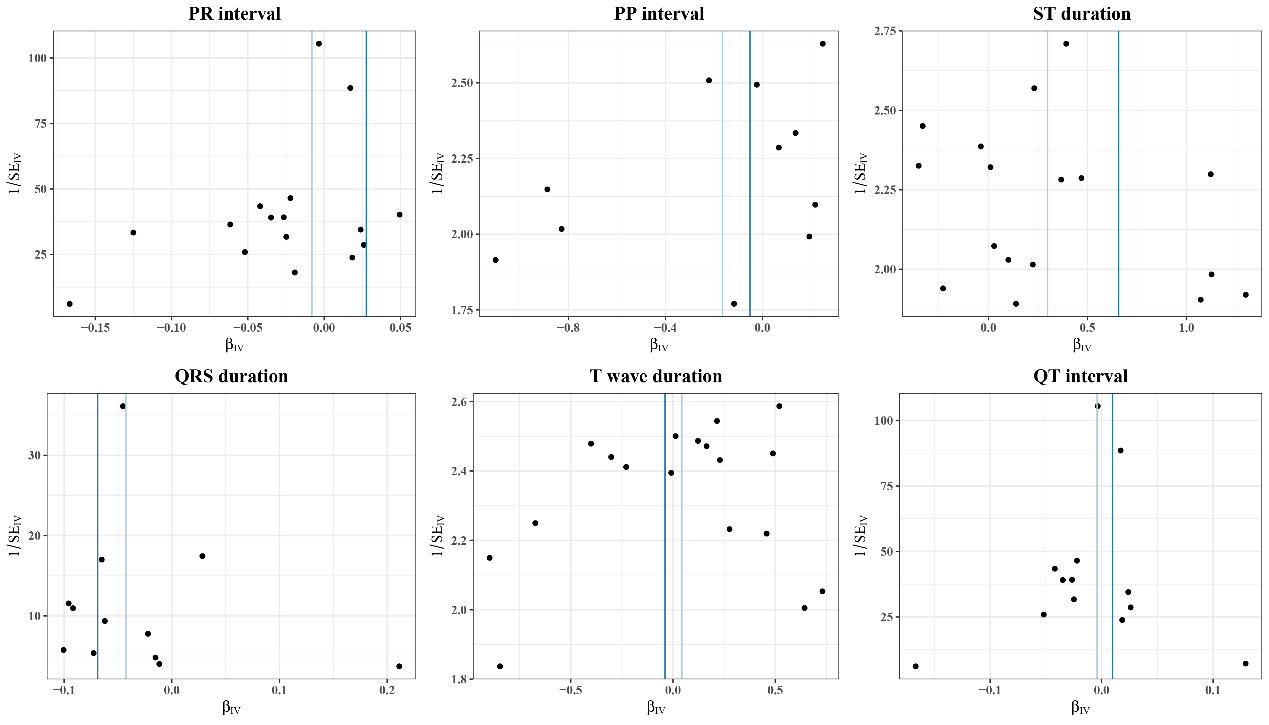
**

**Supplementary Figure 4 The funnel plot for forward MR analysis.**

**
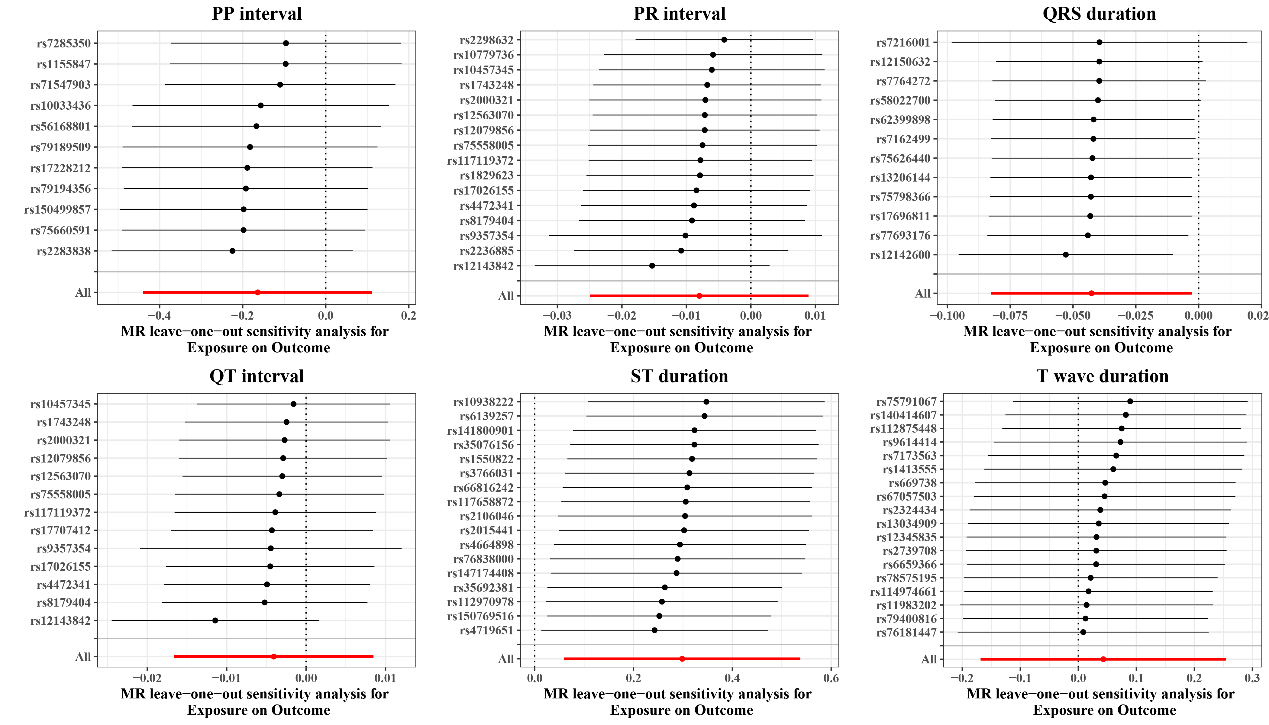
**

**Supplementary Figure 5 The leave-one-out analysis for forward MR analysis.**

**Supplementary table 1 The detail information for all enrolled data in current study**

| **Name** | **Sample Size** | **SNP Count** | **Database** |
| --- | --- | --- | --- |
| PR interval | 13702 | 26496459 | OpenGWAS |
| PP interval | 6693 | 6240610 | OpenGWAS |
| ST duration | 6914 | 6240610 | OpenGWAS |
| QRS duration | 13420 | 26360048 | OpenGWAS |
| T wave duration | 6818 | 6240610 | OpenGWAS |
| P wave duration | 37678 | 842925 | OpenGWAS |
| QT interval | 13698 | 26453374 | OpenGWAS |
| Brugada syndrome | 12821 | 6949690 | OpenGWAS |

**Supplementary table 2 The forward MR analysis for each exposure on BrS**

| **Outcome** | **Exposure** | **Method** | **NSNP** | **Beta** | **SE** | ***P* value** | **OR** |
| --- | --- | --- | --- | --- | --- | --- | --- |
| Brugada | PR interval | Inverse variance weighted | 13 | -0.0040 | 0.0064 | 0.5243 | 0.9959 |
| Brugada | PP interval | Inverse variance weighted | 10 | -0.0957 | 0.1410 | 0.4973 | 0.9087 |
| Brugada | ST duration | Inverse variance weighted | 17 | 0.2984 | 0.1219 | 0.0144 | 1.3478 |
| Brugada | QRS duration | Inverse variance weighted | 12 | -0.0426 | 0.0203 | 0.036 | 0.9582 |
| Brugada | T wave duration | Inverse variance weighted | 18 | 0.0431 | 0.1079 | 0.689 | 1.0440 |
| Brugada | QT interval | Inverse variance weighted | 13 | -0.0040 | 0.0064 | 0.5243 | 0.9959 |

**Notes:** MR, Mendelian randomization; RrS, Brugada syndrome; SE, Standard Error; OR, Odds Ratio; *P* < 0.05 was considered as significant causal relationship

**Supplementary table 3 The reverse MR analysis for each exposure on BrS**

| **Outcome** | **Exposure** | **Method** | **NSNP** | **Beta** | ***P* value** | **OR** |
| --- | --- | --- | --- | --- | --- | --- |
| PR interval | Brugada | Inverse variance weighted | 16 | 2.6174 | 5.9888E-07 | 13.6996 |
| QRS duration | Brugada | Inverse variance weighted | 16 | 0.8472 | 2.3576E-08 | 2.3331 |
| P wave duration | Brugada | Inverse variance weighted | 9 | 1.1662 | 4.8596E-05 | 3.2098 |
| QT interval | Brugada | Inverse variance weighted | 16 | -1.3254 | 2.6967E-06 | 0.2657 |

**Notes:** MR, Mendelian randomization; RrS, Brugada syndrome; OR, Odds Ratio; *P* < 0.05 was considered as significant causal relationship
